# CURRENT TRENDS IN THE DIRECT CARE WORKFORCE

**DOI:** 10.1093/geroni/igae098.3163

**Published:** 2024-12-31

**Authors:** Christopher Kelly, Jerome Deichert

**Affiliations:** University of Nebraska Omaha, Omaha, Nebraska, United States; University of Nebraska Omaha, Omaha, Nebraska, United States

## Abstract

We used data from the 2020-2022 American Community Survey to examine trends among direct care workers (DCWs) in both occupation and industry. We found the number of nursing assistants, psychiatric aides, and home health aides decreased 5.8%, to nearly 1.9 million, while the number of personal and home care aides decreased 0.8%, to more than 1.3 million. Nursing assistants, psychiatric aides, and home health aides were more likely than personal and home care aides to be female, under age 25, African American, recently divorced, and to receive health insurance from their employer. They were less likely to be age 65 and over, to receive health insurance from Medicare or Medicaid, and to have a disability. Nearly 1.9 million DCWs were employed in home and community-based services, as compared to under 900,000 in residential care facilities and under 500,000 in hospitals. There were increases in the number of DCWs employed by outpatient care centers (10.7%), hospitals (7.2%) and individual and family services (3.7%). The largest decreases were in the number of DCWs employed by skilled nursing facilities (17.4%), assisted living facilities (12.3%), and private households (6.1%). DCWs employed by residential care facilities were more likely to be nursing assistants, psychiatric aides, or home health aides and they were more likely to work year-round and full-time. These findings suggest that changes already underway in the direct care workforce accelerated during/after the COVID-19 pandemic. DCWs are shifting towards jobs requiring less training and/or licensure and away from jobs in residential care facilities.

